# An Ultra-Precision Absolute-Type Multi-Degree-of-Freedom Grating Encoder

**DOI:** 10.3390/s22239047

**Published:** 2022-11-22

**Authors:** Shengtong Wang, Linbin Luo, Junhao Zhu, Ningning Shi, Xinghui Li

**Affiliations:** 1 Tsinghua Shenzhen International Graduate School, Tsinghua University, Shenzhen 518055, China; 2 Tsinghua-Berkeley Shenzhen Institute, Tsinghua University, Shenzhen 518055, China

**Keywords:** precision positioning, multi-degree-of-freedom, absolute measurement, grating encoder, synthetic aperture optics, laser autocollimation

## Abstract

An absolute-type four-degree-of-freedom (four-DOF) grating encoder that can simultaneously measure the three-axis pose (*θx*, *θy*, *θz*) and one-axis out-of-plane position (*Z*) of an object with high accuracy is demonstrated for the first time in this research. This grating encoder is composed of a stationary reading head and a movable grating reflector. A light beam from the reading head is projected onto the grating, and three diffracted beams (0th-, +1st-, and −1st-order) are generated, collimated, and received by three separate quadrant photodetectors (QPDs). The information of *θx*, *θy*, *θz*, and *Z* is coded into spot positions of these three diffracted beams on the QPDs. Thus, the modeling and decoupling algorithms were investigated, and an independent calculation of these four-DOF absolute positions was theoretically guaranteed. A prototype was then designed, constructed, and evaluated. Experimental results verified that the proposed grating encoder could achieve the absolute measurement of four-DOF *θx*, *θy*, *θz*, and *Z* with an accuracy of sub-arcseconds and sub-micrometers. To the best of our knowledge, the proposed encoder in this research is the first one to achieve absolute simultaneous measurements of four-DOF position and pose with a large measurement range. The success of this new grating encoder can benefit various multi-DOF positioning applications, especially for large-scale synthetic aperture optics (SAO), including stitching off-axis parabolic mirrors and pulse compression grating.

## 1. Introduction

Large-scale synthetic aperture optics (SAO) systems are highly important in several applications, such as deep space exploration [1], high-energy laser physics, national defense security [2], and other basic research fields [3,4]. The position and pose monitoring of the sub-mirror affects its performance [5]. Therefore, highly accurate, absolute-type, multi-degree-of-freedom position and pose monitoring equipment is necessary for aligning or adjusting each sub-mirror to its ideal posture. 

Currently, the multi-degree-of-freedom position and pose measurement schemes mainly include optical and electrical schemes. The electrical method can demonstrate a high accuracy but is limited in its applications [6,7] because of its short measurement range, the requirement of conductive measurement target, and complexity of expansion to multi-DOF. The optical scheme can provide both a nanometric accuracy and a large range, mainly represented by the laser interferometer [8,9] and the grating encoder [10,11,12,13]. However, the laser interferometer is vulnerable to environmental variation because of its long exposed optical path, which greatly influences the measurement standard, that is, light wavelength. In contrast, the optical path in the grating encoder is much shorter, and its primary measuring standard is the physical grating pitch [14,15,16]. Thus, the grating encoder is less affected by the environment, normally demonstrates a more stable performance, and has great potential for precision position and pose measurement [17,18,19]. However, the state-of-the-art grating encoders mainly focus either on the absolute measurement in one-axis, or on the multi-DOF incremental measurement [20,21,22,23], and there are few types of research on absolute-type multi-DOF measurement, and it remains a large challenge to combine them while keeping a compact and lower weight structure. Thus, there is a pressing need to develop a grating-based 6-DOF absolute position and pose measurement method with high stability, accuracy, and compactness.

To address the same problems, some effective measurement schemes were proposed for in-plane position measurement by phase detection [24]. Li et al. [25,26] proposed an absolute 2-DOF encoder with two probes, which uses the correlation of reference codes to obtain the absolute position information of the measurement target and achieves a 0.5 μm absolute position accuracy in *X*- and *Y*-directions. Furthermore, Shi et al. [27] proposed a hybrid-positioning methodology that combines a pulse signal generated from the correlation of reference codes as that in Ref. [25] and an incremental interference signal to improve the positioning accuracy of the reference position. The positioning repeatability was greatly improved and reached 10 nm for the motion range of several tens of millimeters. Due to the success of the in-plane *X*- and *Y*-direction absolute position measurement, the main research work of the six-DOF absolute measurement can focus on the out-of-plane, i.e., the 4-DOF absolute position and pose measurement of *θx*, *θy*, *θz*, and *Z*-direction. 

Several related types of research have been proposed for out-of-plane measurement [28,29]. Gao et al. first demonstrated a three-DOF (*θx*, *θy*, *θz*,) autocollimator by using a grating reflector to replace the flat mirror in the conventional laser autocollimator [30,31]. Although this innovative autocollimator can provide a high resolution and a simultaneous measurement of three-DOF poses, this is mainly for incremental measurement and not *Z*-direction position measurement. Then, Liu et al. modified the proposal in Ref. [30] and proposed a four-DOF system, allowing simultaneous measurement of four error motions involving *Z*-direction [32], which was then expanded to be five-degrees-of-freedom approach [33]. These studies still focused on incremental measurement tests and did not discuss the possibility of absolute measurement or inevitable alignment error compensation, which is indispensable for these kinds of multi-DOF measurement systems. They also do not demonstrate each posture for the multi-DOF position and pose motion inputs, so these studies lack comprehensive evaluation of decoupling accuracy. The actual experiment ranges in these studies were not sufficient at less than 4 arcseconds and with a large relative measurement error, e.g., the 3×STDEV of yaw error is 0.4′′ in the range of 0.6′′ also prevented its advancement.

In order to meet the high accuracy and absolute measurement demand of the out-of-plane 4-DOFs of the sub-mirror, three improvement works based on the abovementioned multi-DOF out-of-plane research were carried out in this study. Firstly, an absolute zero-point of the QPD coordinate system was proposed to establish the absolute coordinate of the spot position, and the absolute position and pose of the grating reflector can be decoupled by the absolute coordinates of the three diffracted spots (+1st-, 0th-, and −1st-order beams). Secondly, a homogeneous error compensation matrix involving installation posture error and installation distance error was proposed, which can significantly reduce the crosstalk error and improve the measurement accuracy. Finally, a compact prototype system was designed and built in this study. For the first time, a verification experiment of simultaneous input of multiple main motions was used to obtain the optimal homogeneous error compensation matrix, and high-precision independent decoupling of the absolute position and pose of the out-of-plane 4-DOF was realized, fully verifying the excellent performance of the proposed grating encoder.

## 2. Principle and Method

**Figure 1:**
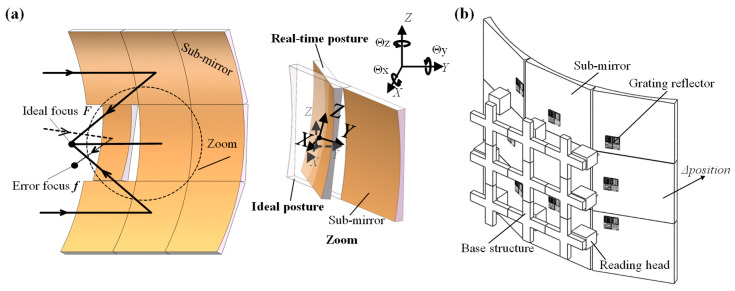
(**a**) The focus change of the synthetic aperture optics (SAO) and the ideal and real-time postures of the sub-mirror; (**b**) the application of the encoder.

As shown in Figure 1a, the change in the SAO focus and the error between the ideal and real-time posture of the sub-mirror decreases the SAO performance. The proposed encoder is used as a monitor in the system described in Figure 1b to help the actuator to adjust the sub-mirror to its ideal posture.

**Figure 2:**
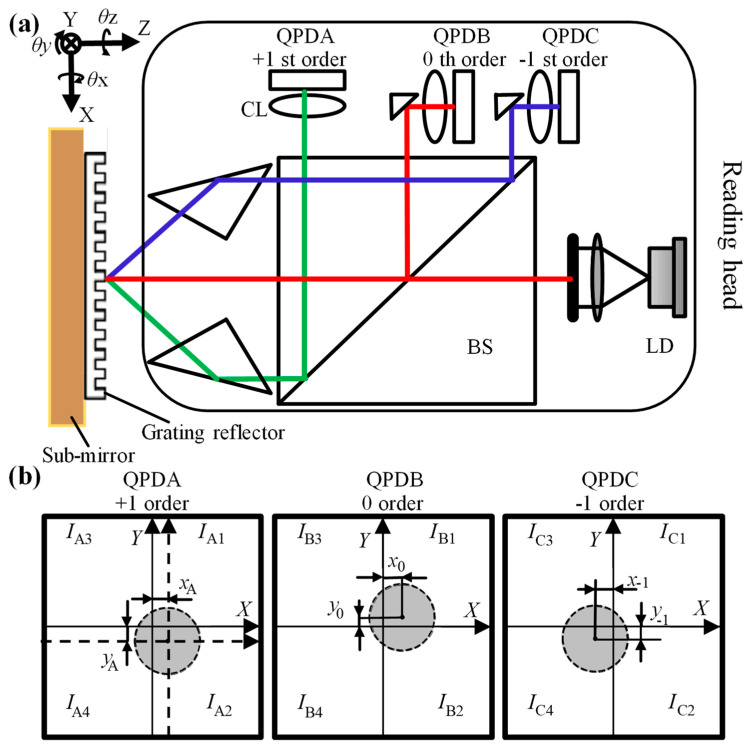
(**a**) The schematic of the encoder; (**b**) the spot coordinate of the QPDs.

Figure 2a shows the schematic of the grating encoder. This grating encoder is composed of a stationary reading head and a movable grating reflector. The 660 nm wavelength laser beam is emitted from the laser diode (LD) and illuminates the grating reflector through the beam splitter (BS). The diffraction beams are the +1st-, 0th-, and −1st-order beams, which are refracted and focus on three QPDs through three convex lenses (CLs). The position information regarding the light spot on the QPD is shown in Figure 2b. The specific positions (*x*_A_, *y*_A_, *x*_B_, *y*_B_, *x*_C_, *y*_C_) of the light spot are calculated according to the back-end photocurrent information, and the specific calculation formula can be expressed as formula (1). In this paper, the obtained coordinates are absolute coordinates; thus, the position coordinates on the QPD are all signed, and from the front of the QPD, right and up are positive directions.

(1) $$\{\begin{array}{l} x_{\alpha }=\frac{\left(I_{\alpha }{}_{1}+I_{\alpha }{}_{2}\right)-\left(I_{\alpha }{}_{3}+I_{\alpha }{}_{4}\right)}{I_{\alpha }{}_{1}+I_{\alpha }{}_{2}+I_{\alpha }{}_{3}+I_{\alpha }{}_{4}},\\ y_{\alpha }=\frac{\left(I_{\alpha }{}_{1}+I_{\alpha }{}_{3}\right)-\left(I_{\alpha }{}_{2}+I_{\alpha }{}_{4}\right)}{I_{\alpha }{}_{1}+I_{\alpha }{}_{2}+I_{\alpha }{}_{3}+I_{\alpha }{}_{4}}, \end{array}$$

where *I*_α_ are the four-way current signal outputs of the QPD, *α* = A, B, or C.

Taking QPDA as an example, due to different light intensity distributions, the coordinate value can be calculated from Formula (1). The coordinate origin of the two-dimensional coordinate system is the symmetric center of the four photodetectors. Due to the mechanical structure, it is difficult to adjust the light spot to be perfectly located at the symmetrical center of the QPD. Therefore, at the beginning of the test, the light spot is located at the position (*x*_A_, *y*_A_) of the coordinate system of the QPD, and *x*_A_ and *y*_A_ are very close to 0. The point (*x*_A_, *y*_A_) is used as the origin of a new QPDA coordinate system in Figure 2b, which is represented in the form of the dashed line. In the new QPD coordinate system, the light spot is located at the coordinate origin, The new coordinate systems of QPDB and QPDC are simultaneously established according to this method.

First, the initial positions of the spots are set as the zero-point positions, i.e., every spot position is at the origin of the new QPD coordinate system. When the grating moves simultaneously in four degrees of freedom, the three light spots in the QPDA, QPDB, and QPDC assume the (*x*_1_, *y*_1_), (*x*_0_, *y*_0_), and (*x*_−1_, *y*_−1_) positions, respectively, which are unique coordinates for calculating the absolute position and pose. 

In these coordinates, the position of the 0th-order light change is only related to θ*x* and θ*y*, which can be solved in Formulas (2) and (3). In addition, *z* and θ*z* also cause the movement of the other spots and can be solved using Formulas (4) and (5).

(2) $$\theta_{x}=\frac{k_{\theta_{x}}y_{0}}{2f}=k_{\theta xz1}\frac{k_{\theta_{x}}y_{1}}{2f}=k_{\theta xz-1}\frac{k_{\theta_{x}}y_{-1}}{2f},$$

(3) $$\theta_{y}=\frac{k_{\theta_{y}}x_{0}}{2f}=k_{\theta ydz1}\frac{k_{\theta_{y}}x_{1}}{2f}=k_{\theta ydz-1}\frac{k_{\theta_{y}}x_{-1}}{2f},$$

(4) $$\theta_{z}=k_{\theta z}\frac{\left(y_{-1}-y_{0}/k_{\theta_{xz-1}})-(y_{1}-y_{0}/k_{\theta_{xz1}}\right)}{2L},$$

(5) $$z=k_{z}\frac{x_{-1}-x_{0}/k_{\theta ydz-1}+x_{1}-x_{0}/k_{\theta ydz1}}{2},$$

where *k_z_*, *k*_*θx*_, *k*_*θy*_, and *k*_*θz*_ are the parameters measured in the pre-experiment. *f* is the focal length of the convex lens, and *L* is the equivalent distance between the photodetectors A and B (or C and B). *k_θxz_*_1_ and *k_θxz_*_−1_ are the impact factors of θ*x* on the +1st- and −1st-order spot positions during the movement, and so are *k_θydz_*_1_ and *k_θydz_*_−1_ of θ*y*.

However, in the simultaneous motions of 4-DOF absolute position and pose experiment, there is an unexpected posture error inevitably generated between the grating encoder measured value ***O*** (original value, shown in Formula (6)) and the true value ***T*** (shown in Formula (7)) due to the imperfection in grating encoder alignment. This posture error can be viewed as a system error and be predicted by using a compensation matrix. The error compensation matrix is a 4 × 4 matrix (***M***), which can be obtained using Formula (8) after the pre-experiment. With this error compensation matrix, the original value can be converted into the output of the grating encoder (*θx*_Encoder, *θy*_Encoder, *θz*_Encoder, *z*_Encoder).

(6) $$O=\left[\begin{array}{ccc} \theta x_{1O\_main} & \theta x_{2O} & \theta x_{3O}\\ \theta y_{1O} & \theta y_{2O} & \theta y_{3O}\\ \theta z_{1O} & \theta z_{2O\_main} & \theta z_{3O}\\ z_{1O} & z_{2O} & z_{3O\_main} \end{array}\right],$$

(7) $$T=\left[\begin{array}{ccc} \theta x_{1T\_main} & \theta x_{2T} & \theta x_{3T}\\ \theta y_{1T} & \theta y_{2T} & \theta y_{3T}\\ \theta z_{1T} & \theta z_{2T\_main} & \theta z_{3T}\\ z_{1T} & z_{2T} & z_{3T\_main} \end{array}\right],$$

***M*** × ***O*** = ***T***, (8)

where ***M*** can be obtained in the pre-experiment with one main motion.

Due to installation errors, the 4-DOF movement is output simultaneously. The three main motions move separately, i.e., *θx*, *θz*, z, thus generating three groups of main and error motions. Then, A matrix ***O*** composed of *θx*_Original, *θy*_Original, *θz*_Original, and *z*_Original can be collected, and ***M*** = ***T*** × ***O***^−**1**^.

## 3. Experiments and Discussion

**Figure 3:**
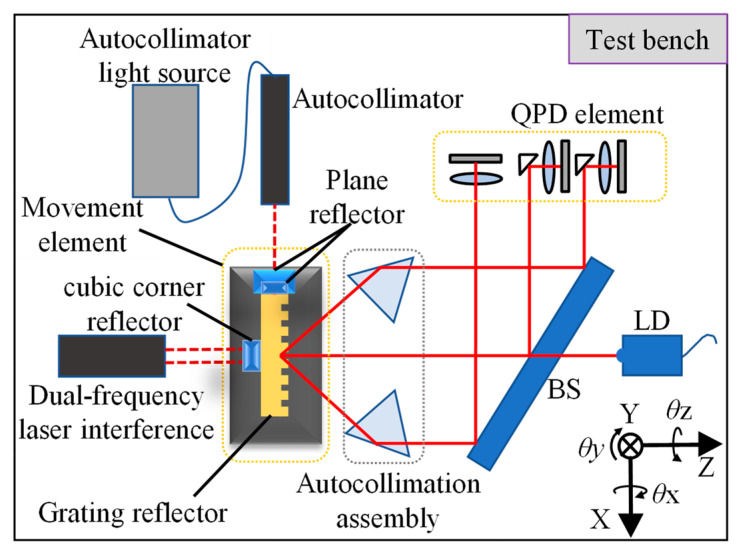
Schematic figure of test bench.

The absolute 4-DOF grating encoder test bench was built, and the schematic figure of the experiment is shown in Figure 3. The measurement truth value is mainly obtained by the dual-frequency laser interferometer and the autocollimator. A reflector is placed below the grating at 45 degrees, and a plane reflector is pasted at the grating so that the laser autocollimator can measure *θx* and *θz*.

**Figure 4:**
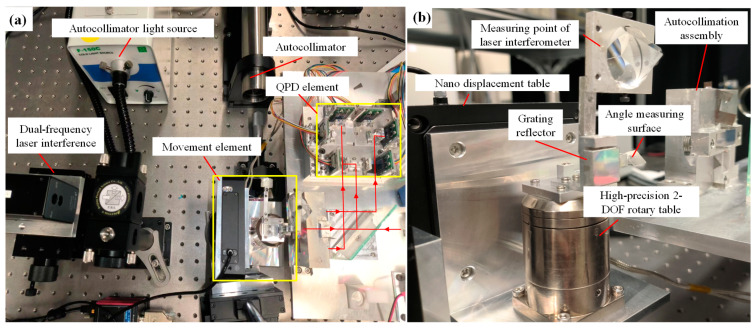
(**a**) The test bench; (**b**) movement element.

The actual test bench is shown in Figure 4a. The autocollimator can measure the absolute angle (*θx*, *θz*) of the grating with a measurement accuracy of 0.1′′. The laser interferometer can measure the absolute displacement of the z-direction with a resolution and measurement accuracy of 0.1 nm and ±160 nm, respectively. The movement element is shown in Figure 4b, which can give the main motions of *θx*, *θz*, *z*, and error motion *θy*. The z-direction movement is controlled by the high-dynamic Z Nanopositioner (PI) (model: p-733. Z), with a resolution of 0.3 nm and a linearity of 0.03%, and the angles are controlled by a two-axis tilt table driver, with a resolution of 0.02” and a linearity of 0.5%.

The value measured by the laser interferometer and autocollimator is called the true value, as described below and shown in the following figure as *θx*_True, *θz*_True, and *z*_True. Owing to the limitation of the autocollimator, the true value of *θy* was obtained during another set of repeated experiments by changing the measurement surface. The following single motion, resolution, and multiple motion experiments were carried out in this study.

**Figure 5:**
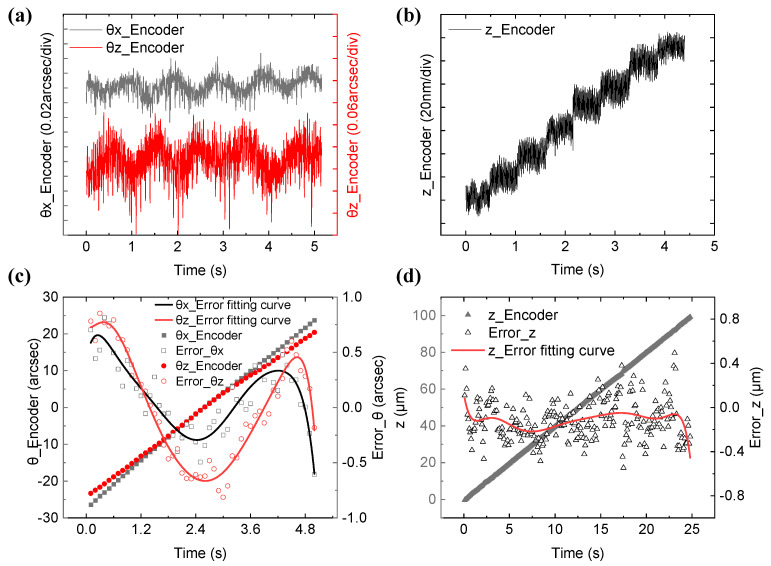
(**a**) Angle resolution of the encoder; (**b**) displacement resolution of the encoder; (**c**) angle error between the encoder value and the true value in a single degree; (**d**) the displacement error between the encoder value and the true value in a single degree.

The pre-experiment was performed in *θx*(80′′), *θz*(80′′), and the z-direction (100 μm) as the main motions and obtained all the constant parameters and the error compensation matrix (***M***) in Formulas (2)–(8). As is evident from Figure 2b, it is difficult to adjust the spot center to the QPD center, but when the spot is located at the center of the QPD, the measurement sensitivity and the resolution of the encoder is the highest. In this study, the distance between the spot center and the QPD center is represented by the coefficient of variation. The coefficients of variation, i.e., the ratio of standard deviation to average of *I*_α1_, *I*_α2_, *I*_α3_, and *I*_α4_ on QPDs A, B, and C are 0.1802, 0.2662, and 0.3135, respectively. Upon this, the resolutions of rotation around the x- and z-axes and the displacement along the *z*-axis are 0.02′′, 0.06′′, and 20 nm, respectively, as shown in Figure 5a,b. Thus, the coefficient of variation values confirm that the encoder’s resolution and accuracy can be further improved, and it can work with high accuracy after being disturbed in practical use.

In Figure 5c,d, when a single freedom motion is provided by the movement element in 5 s, the residual errors are −0.60 to 0.70 arcsec in 50 arcsec *θx* rotation, −0.81 to 0.85 arcsec in 40 arcsec *θz* rotation, and −0.55 to 0.49 μm in the 100 μm z-direction displacement. This indicates that in the continuous measurement of a single degree of freedom, the encoder has good linearity and stability without a large drift.

**Figure 6:**
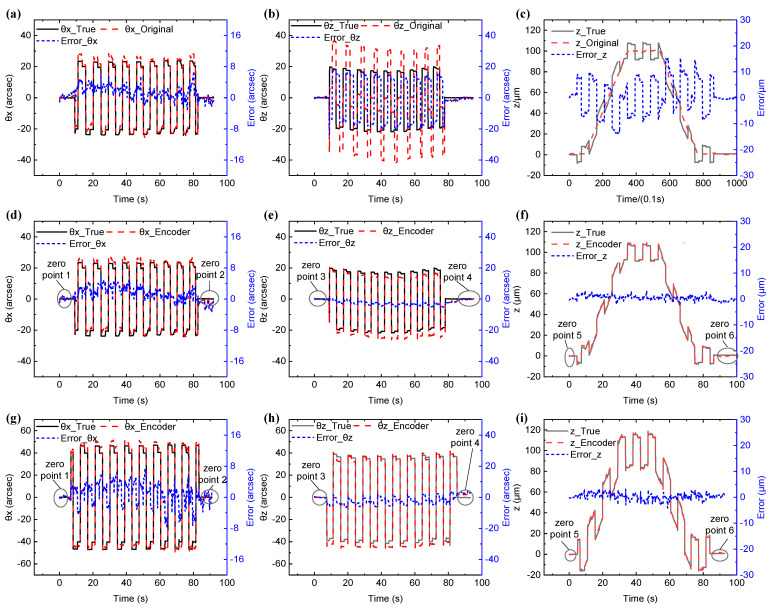
(**a**–**c**) Position and pose errors without compensation; (**d**–**i**) position and pose errors after compensation.

In multiple motion experiments, the square wave rotation motions with a frequency of 0.1 Hz and the amplitude of 40′′ and 80′′ around the x- and z-axes and a z-direction motion with 100 μm at a speed of 2 μm/s are simultaneously given by the movement element. Three main movements are input concurrently, and their comparison with the true value is shown in Figure 6. Since the movement element is a square wave motion, there are data points during an abrupt change, and the original value cannot align with the true value on the timeline. Therefore, the data areas that rise or fall steadily were selected for data comparison, and the length of time was also changed in one simultaneous test. Since the movement element’s accuracy is considerably high, this comparison method can characterize the measurement performance of the encoder.

Because the measurement point of the *z*-axis displacement is different from that of the grating encoder in periodic motion, a moving arm length linearly increases the displacement along the *z*-axis. Since the error matrix calculation includes the linear relationship based on this structure, the measured value of the laser interferometer represents the true value of the grating displacement herein.

During the period (*θx* = 40′′, *θz* = 40′′, *z* = 100 μm) shown in Figure 6d–f before the error matrix compensation, the grating encoder’s original value is stable but not correct. From the output of the encoder’s original signal, because of the simultaneous movement of multiple degrees of freedom, the angular measurement error around the *x*-axis is about −3′′ to 7′′, while the angular measurement error around the *z*-axis is large, reaching the degree of about ±40′′. Additionally, the angular measurement error first decreases overall and then rises and returns, mainly because the rotation around the *z*-axis is affected by *z*-axis displacement. The error of the original value is far from the true value; therefore, the original signal requires compensation by the error matrix.

Figure 6d–i show that in the output after compensation, the measurement error is greatly reduced. When the motion period (*θ*x = 40′′, *θ*z = 40′′, *z* = 100 μm) is applied to the grating, the *θz* error range is (−23.58′′, 18.01′′) before compensation and (−5.56′′, 0.98′′) after compensation. The error range in z-direction position is (−13.64, 13.94 μm) before compensation and (−2.33, 3.26 μm) after compensation. When the motion period (θ*x* = 80′′, θ*z* = 80′′, *z* = 100 μm) is applied to the grating after compensation, the *θz* error range is (−6.52′′, 3.91′′), and the error range in the z-direction position is reduced to (−4.13, 3.42 μm). *θx* remains a relatively low error range without obvious change in these two periods. During the period (*θx* = 40′′, *θz* = 40′′, *z* = 100 μm) shown in Figure 6d–f, the root mean square (RMS) of the errors of the encoder value relative to the true value is shown in Table 1. The standard error around the *x*-axis increased by 2.26%, mainly because the fluctuations of the other three degrees of freedom were superimposed on it after compensation by the error matrix. The RMS of the errors around the *z*-axis was reduced by 78.99%, and the RMS of the errors of the displacement of the *z*-axis was reduced by 88.14%, demonstrating the effectiveness of the error matrix compensation method. As shown in Figure 6g–i, when the period is *θx* = 80′′, *θz* = 80′′, and *z* = 100 μm, the same compensation matrix is used, and the RMS of the errors of the encoder value is 3.02′′, 3.25′′, and 1.21 μm in *θx*, *θz*, and *z*-direction, respectively. When the measuring range is enlarged, the error does not increase obviously, which proves the effectiveness and reliability of the compensation method.

Table 2 shows the average residual error of the zero-point of the absolute four-degree encoder with a different motion period. The encoder is stable in the sub-arcsecond and sub-micron when returning to the origin of position and pose in *θx* and *z*-direction. Initially, the precision of sub-angular seconds can be reached in *θz*; however, after a movement of long duration, the average measurement residuals of −1.03′′ and 2.93′′ are generated, respectively. This is because in the calculation of the roll angle of the grating, the rotation movement of the light spot is simplified to a linear movement, and its initial position is not at the QPD center, resulting in the enhancement of nonlinearity and error during the measurement. 

**Figure 7:**
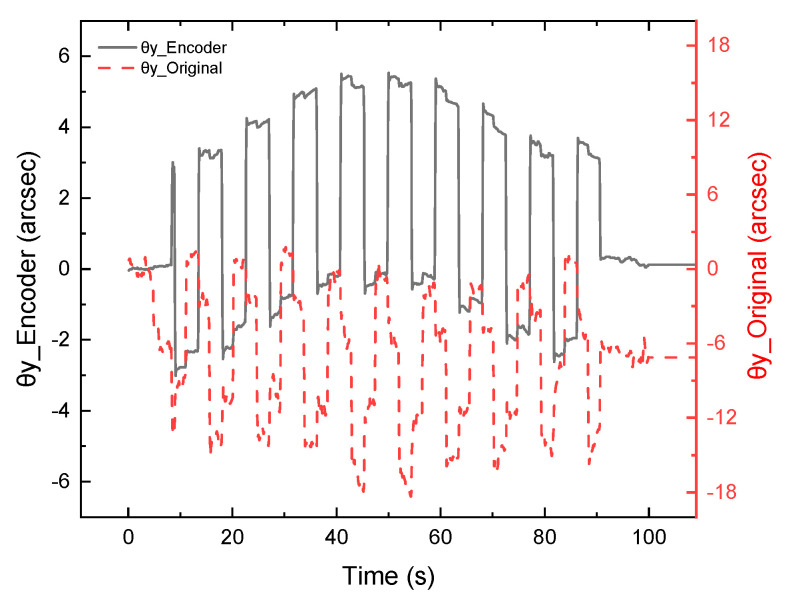
The *θy* value (40′′40′′100 μm) comparison between the original value and the value after compensation.

Since the autocollimator cannot measure 3-DOF angles simultaneously, only the compensation data are shown in Figure 6. The error motion of *θ_y_* is not caused by crosstalk but by the motion of the movement element itself due to the installation, which could be detected in the repeated experiments. The data time length in Figure 7 is different from that of Figure 6 as it does not align the time with the true value and retains the original value, which is highly unstable. After the compensation, *θ_y_* is more stable and shows a similar change of displacement of the *z*-axis, indicating that the compensation matrix method is correct. It proves that during the simultaneous operation of the four degrees of freedom, although the DOFs affect each other, they share a relatively linear relationship within a certain range. A larger measurement range and accuracy can be achieved by optimizing the nonlinear formula and the design of the optical path onto the QPD center. 

## 4. Conclusions

This paper proposes an ultra-precision absolute-type multi-degree-of-freedom grating encoder. Three aspects are studied as follows: Firstly, the absolute position matrix was constructed by establishing the zero-points of the diffracted light spots on QPD coordinates, and the absolute 4-DOF position and pose (*θx*, *θy*, *θz*, and *Z*) of the grating reflector in space can be decoupled. Secondly, the homogeneous error compensation matrix was proposed to greatly reduce the crosstalk error caused by installation posture error and installation distance error and improve the accuracy. Finally, a compact prototype system was designed, and the effectiveness of the proposed structure and compensation matrix was fully verified by experiments. Experiments demonstrate that this encoder can provide absolute 4-DOF position and pose monitoring with sub-arcsecond and sub-micron accuracy and high stability. 

In terms of absolute measurement, the measurement of *x* and *y* can be realized at present [25,26,27], but the absolute measurement of the other four degrees of freedom cannot. The measurement schemes [30,31] do not discuss the possibility of absolute measurement and only refer to the increment measurement of three angles, which excludes the measurement of *z*-direction. The current commercial products, such as the TriAngle Products of *Trioptics* [34], can only realize the measurement of two angles. Therefore, the encoder proposed in this paper is very meaningful in the field of absolute multi-degree of freedom measurement and has great application prospects in future precision positioning. 

## Figures and Tables

**Table 1:** The RMS of the error of the encoder value relative to the true value.

Error Value	Original Value	After Compensation	Error Change Percentage/%
θ*x*/(′′)	2.06	2.11	+2.26
θ*z*/(′′)	13.24	2.78	−78.99
*z*/(μm)	8.66	1.03	−88.14

**Table 2:** Encoder average residual of the zero-point.

Zero-Point	1/2(′′)	3/4(′′)	5/6(μm)
40′′40′′100μm	0.10/−1.46	−0.21/−1.03	0.0002/−0.73
80′′80′′100μm	−0.07/−0.27	−0.14/2.93	0.10/0.62

## Data Availability

The data presented in this study are available on request from thecorresponding author.
